# The impact of perceptual complexity on road crossing decisions in younger and older adults

**DOI:** 10.1038/s41598-023-49456-9

**Published:** 2024-01-04

**Authors:** Victoria I. Nicholls, Jan Wiener, Andrew Isaac Meso, Sebastien Miellet

**Affiliations:** 1 Department of Psychology and Sports Sciences, Goethe University, Frankfurt, Germany; 2 Department of Psychology, Bournemouth University, Poole, UK; 3 Neuroimaging Department, King’s College London, London, UK; 4 Department of Psychology, University of Wollongong, Wollongong, Australia

**Keywords:** Psychology, Human behaviour

## Abstract

Cognitive abilities decline with healthy ageing which can have a critical impact on day-to-day activities. One example is road crossing where older adults (OAs) disproportionally fall victim to pedestrian accidents. The current research examined two virtual reality experiments that investigated how the complexity of the road crossing situation impacts OAs (N = 19, ages 65–85) and younger adults (YAs, N = 34, ages 18–24) with a range of executive functioning abilities (EFs). Overall, we found that OAs were able to make safe crossing decisions, and were more cautious than YAs. This continued to be the case in high cognitive load situations. In these situations, safe decisions were associated with an increase in head movements for participants with poorer attention switching than participants with better attention switching suggesting these groups developed compensation strategies to continue to make safe decisions. In situations where participants had less time to make a crossing decision all participants had difficulties making safe crossing decisions which was amplified for OAs and participants with poorer EFs. Our findings suggest more effort should be taken to ensure that road crossing points are clear of visual obstructions and more speed limits should be placed around retirement or care homes, neither of which are legislated for in the UK and Australia.

## Introduction

The gradual decline of cognitive abilities is a normal part of healthy ageing. This decline can have a critical impact on day-to-day activities. One of these activities is that of road crossing where older adults (OAs) disproportionally fall victim to pedestrian accidents compared to other age groups^1,2^. Road crossing is a common task that needs high levels of bodily and neurological coordination, it requires body, head, and eye coordination, integration of information about moving targets from different parts of the visual fields, and fast decision making, which has potentially dire consequences should any of these processes fail. A number of these processes, known as executive functioning (EF), decline with age. Previous studies have shown that OAs with declining EFs and flawed visual sampling strategies make more unsafe crossing decisions^3–5^. Typically, these studies look for a general decline in road crossing performance and only manipulate one or two aspects of the crossing task. What remains to be examined is a systematic manipulation of the road crossing task to determine which aspects of road crossing OAs have difficulties with and which cognitive factors are linked to these different road crossing aspects.

Previously, we investigated the effect of ageing, independently of any decline in EF, on visual attention and decision making during road crossing^6^. We found that OAs with maintained EFs showed similar overt attention strategies and made similar road crossing decisions to younger adults (YAs). We found that environmental constraints, EF variations within a healthy range, and ageing impact on how the road crossing task was performed. OAs crossed when gaps between cars were larger and safer which was amplified in high cognitive load conditions. Our interpretation is that these conservative decisions are strategies used to maintain safe crossing behaviours when the road crossing situation is more challenging.

However, the OAs’ EF level was in the range of YAs, thus preventing us from directly studying the effects of a decline of EFs on road crossing performance. Moreover, the video stimuli presented had only one traffic direction and one lane of traffic with a limited field of view. The stimuli were ambient videos so there was no a-priori experimental control of the vehicles’ speed, the number and density of vehicles, and the number and characteristics of distractors. Consequently, the task was relatively easy, allowing for successful compensatory strategies (waiting for larger crossing gaps). Even so, we found limits to these compensatory strategies. Poorer attention switching abilities combined with age related differences in visual sampling strategies led to reduced attention switches toward initial vehicle movements, suggesting that older individuals with poor attention switching abilities could have difficulties in early scanning of vehicles and predicting their trajectories. This could lead to riskier crossing decisions when cognitive loads are high, for example when having cars travel from multiple directions at once.

Furthermore, working memory (WM) decline in OAs^7–9^ may cause additional problems in situations where cars travel from multiple directions. OAs may not be able to store and use all the vehicle trajectories coming from the different directions in order to make safe crossing decisions. However, in situations where cars only travel from one direction or on one lane less information is required to be held in WM, making it easier for OAs to make safe decisions. Thus, OAs may still be able to perform at the same level as YAs in these simpler situations^6^, but in more complex situations OAs would have difficulties. Consequently, we anticipate that if we introduce more intricate road crossing scenarios involving cars coming from multiple directions and higher traffic density, OAs will make less safe crossing decisions. Specifically, they may attempt to cross when cars are dangerously close to them.

Alongside situations with high WM load, OAs may find it difficult to make safe crossing decisions when cars are travelling at high speeds. OAs have been shown to have slower visual processing speeds than YAs^10–13^. Therefore, OAs may not be able to take in enough information about vehicles’ trajectories to be able to make safe crossing decisions when the cars are travelling quickly. However, in situations where cars are travelling slowly, OAs may still be able to make safe crossing decisions despite slower processing speeds, as they have more time to take in information about the vehicles and the crossing situation in general. We, therefore, predict that when cars travel quickly OAs will make less safe crossing decisions, perhaps by choosing to cross when the cars are too close to the participant.

Indeed there is evidence that increasing the complexity of the crossing task, through cars travelling from both directions^14^, from the far lane^5,14,15^, performing another task alongside road crossing (talking and crossing;^16^) or cars travelling quickly reduce OAs ability to make safe crossing decisions^3,15,17^.

Building on this there is also evidence that a decline in EFs such as spatial planning and attention switching is associated with making less safe crossing decisions^5^. In a virtual reality experiment, Bock et al.^18^ found that each glance at a traffic light took longer in the older group and may reflect a generalised slowing of visual processing in OAs. It could also be a sign of OAs having problems with suppressing automated saccades towards irrelevant objects^19–24^. Furthermore, Zito et al.^4^ linked visual sampling, EFs, and road crossing decisions with age. They showed that OAs spend more time than YAs looking at the ground in front of them which was linked to more unsafe crossing decisions and a decline in EF.

These studies have separately addressed the effect of specific traffic situations, such as cars travelling from both directions, and the effect of executive functioning, or the combined effect of car speed and a decline in executive functioning on the ability to make safe crossing decisions. None of them have examined the combined effect of declining executive functioning with systematic increase in task complexity on road crossing performance.

In this study we examined how the complexity of the road crossing situation impacts OAs and YAs with a range of EFs. As YAs are involved in the fewest number of accidents of any age group^1,2^ we assume that they make the most ideal crossing decisions, so would make a suitable control group to compare OAs crossing decisions against. We performed two experiments to investigate the impact of increased perceptual and cognitive complexity on OAs road crossing performance. Before the road crossing tasks, the participants EF abilities and walking speed were measured. Both experiments used virtual reality (VR) scenes to allow for a precise control of the experimental conditions and a wide visual field (180°). The first experiment examined whether the number of lanes (one vs two), the lane used (near vs far lane), car speed, and car view impacted performance. The second experiment assessed the impact of cars travelling in both vs one direction, the presence of pedestrian distractors, the vehicles’ speed, traffic density, and how early the vehicles were visible on crossing performance.

## Introduction: Experiment 1

Experiment 1 was performed to resolve two confounds in one of our road crossing situations—cars travelling from both left and right directions. Typically, when cars travel from two directions they travel along two lanes, however, when they travel from one direction, they typically travel from one lane. Therefore, any effects that might be associated with cars travelling from two directions instead of one cannot be separated. Moreover, cars travelling from both directions are travelling along both the near and the far lane, while cars travelling from one direction are travelling along the near or the far lane. Therefore, any effects of cars travelling along the far lane^5,14,15^ cannot be separated from cars travelling along both lanes at once^14^. To resolve this, we examined crossing behaviour in OAs and YAs when cars travelled along one lane compared to two lanes, as well as cars travelling in the near lane, far lane, and two lanes.

In this experiment we also manipulated the speed of the cars and the view of the cars to determine whether these conditions would still have an impact on crossing decisions even when the crossing task was relatively simple (in comparison to Experiment 2).

## Results: executive function tests

**Figure 1:**
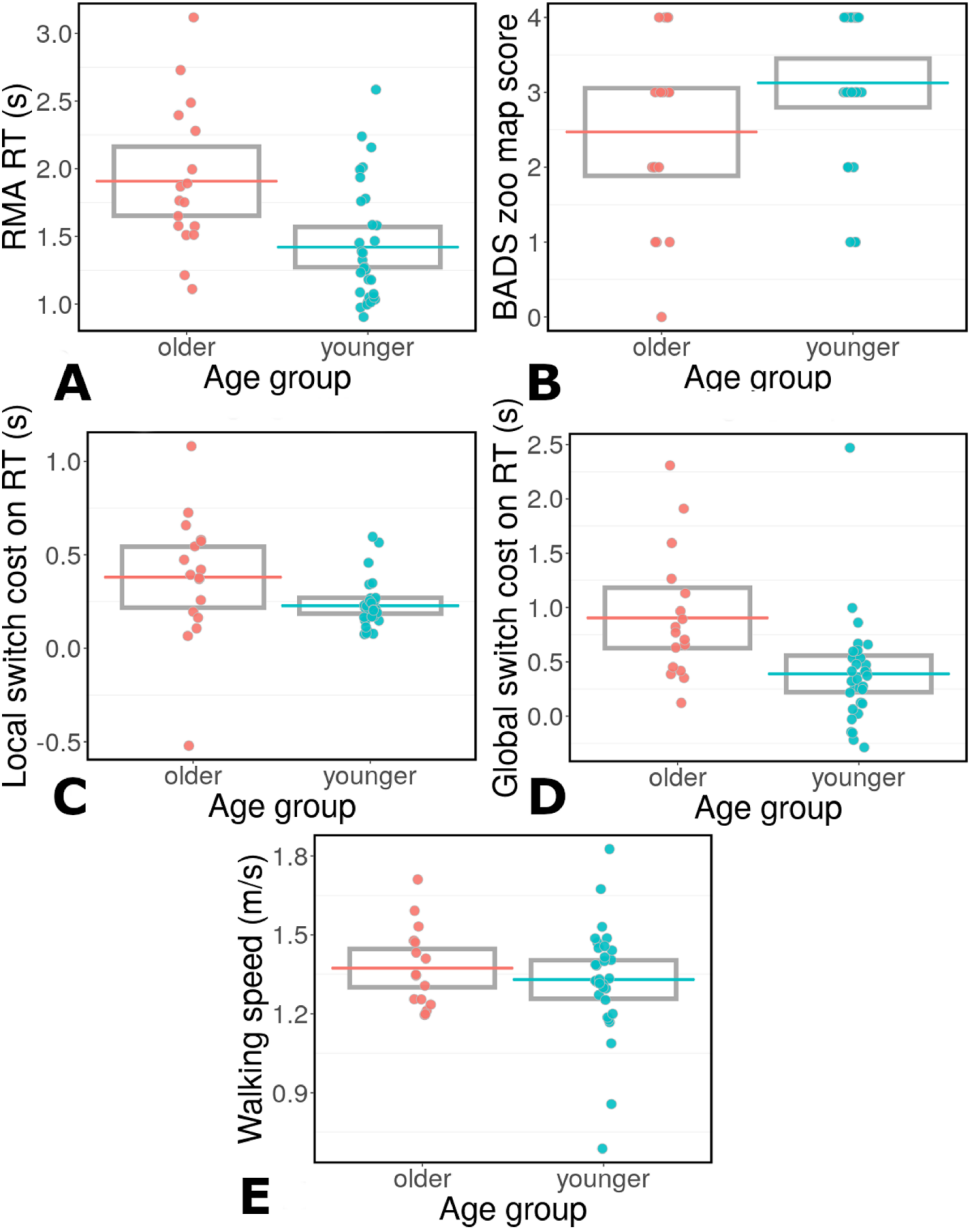
Executive function results. Participants’ RTs (**A**) on the RMA task. Local switch costs on RMA task RT (**C**). Global switch costs on RMA task RT (**D**). Participants’ BADS zoo map scores (**B**). Participants’ walking speed (**E**). In all panels the red colours indicate OAs and blue colours indicate YAs.

Bootstrapped t-tests and Bayes factors indicated that OAs and YAs had similar walking speeds (Table 1, Fig. 1E), BADS zoo map scores (Table 1, Fig. 1B). Older adults showed significantly longer response times on the RMA task than YAs (Table 1, Fig. 1A), as well as larger local and global switch costs on the RMA task than YAs (Table 1, Fig. 1C,D respectively).

**Table 1:** Means, bootstrap t-tests, and Bayes Factors for the differences between OAs and YAs for the different EF measures, testing attention switching ability (local and global switch costs and RMA response times), spatial planning (BADS zoo map test), and walking speed.

	Means	SE	t-vale	df	CIs	p-value	d	Bayes Factor
Walking speed	YA: 1.33 OA: 1.37	YA:0.04 OA:0.04	0.12	17.51	[− 0.10, 0.12]	0.903	0.08	0.37
BADS	YA: 3.10 OA: 2.63	YA:0.17 OA:0.27	− 1.68	12.25	[− 1.77, 0.16]	0.099	0.42	1.63
RMA RT	*YA: 1.42 OA: 1.93*	*YA:0.08* *OA:0.14*	*3.05*	*17.36*	*[0.17; 0.85]*	*0.005*	*0.64*	*27.29*
Local switch cost	*YA: 0.23 OA: 0.38*	*YA:0.02* *OA:0.09*	*2.68*	*10.79*	*[0.03; 0.34]*	*0.022*	*0.56*	*2.25*
Global switch cost	*YA: 0.40 OA: 0.94*	*YA:0.09* *OA:0.15*	*2.98*	*14.39*	*[0.14; 0.74]*	*0.008*	*0.70*	*18.17*

Significant results are italics.

## Results Experiment 1

There were no significant effects of cars coming from two lanes compared to one lane; or cars travelling in the near versus far lane on TTI (Table 2, and Supplementary Table S1).

**Table 2:** LMM results for the TTI left by participants in Experiment 1.

Factor	β	SE	t-value	p-value
Car speed	− *1.80*	*0.70*	− *2.58*	*0.010*
Car speed * BADS	*0.41*	*0.13*	*3.06*	*0.002*
Car speed * Age * Global switch cost	*2.27*	*0.93*	*2.45*	*0.014*
View	− *2.09*	*0.68*	− *3.07*	*0.002*
View * Local switch cost	*2.80*	*1.42*	*1.97*	*0.049*
View * BADS	*0.33*	*0.13*	*2.53*	*0.011*
View * Age * Local switch cost	− *3.29*	*1.54*	− *2.14*	*0.033*
BADS	− *0.97*	*0.25*	− *3.85*	*0.0003*
Lane number	− 0.03	0.83	− 0.03	0.974
Near/Far Lane	− 0.57	0.81	− 0.71	0.480

Significant results are highlighted in italics. Only significant main effects and interaction effects for car speed, view of the cars, age, BADS, local and global switch costs, are listed. Number of lanes and lane type (1 or 2) factors are listed as these are the main factors of interest for Experiment 1. For full LMM results see Table 1 in the Supplementary Materials. See Methods section for the models that were run.

**Figure 2:**
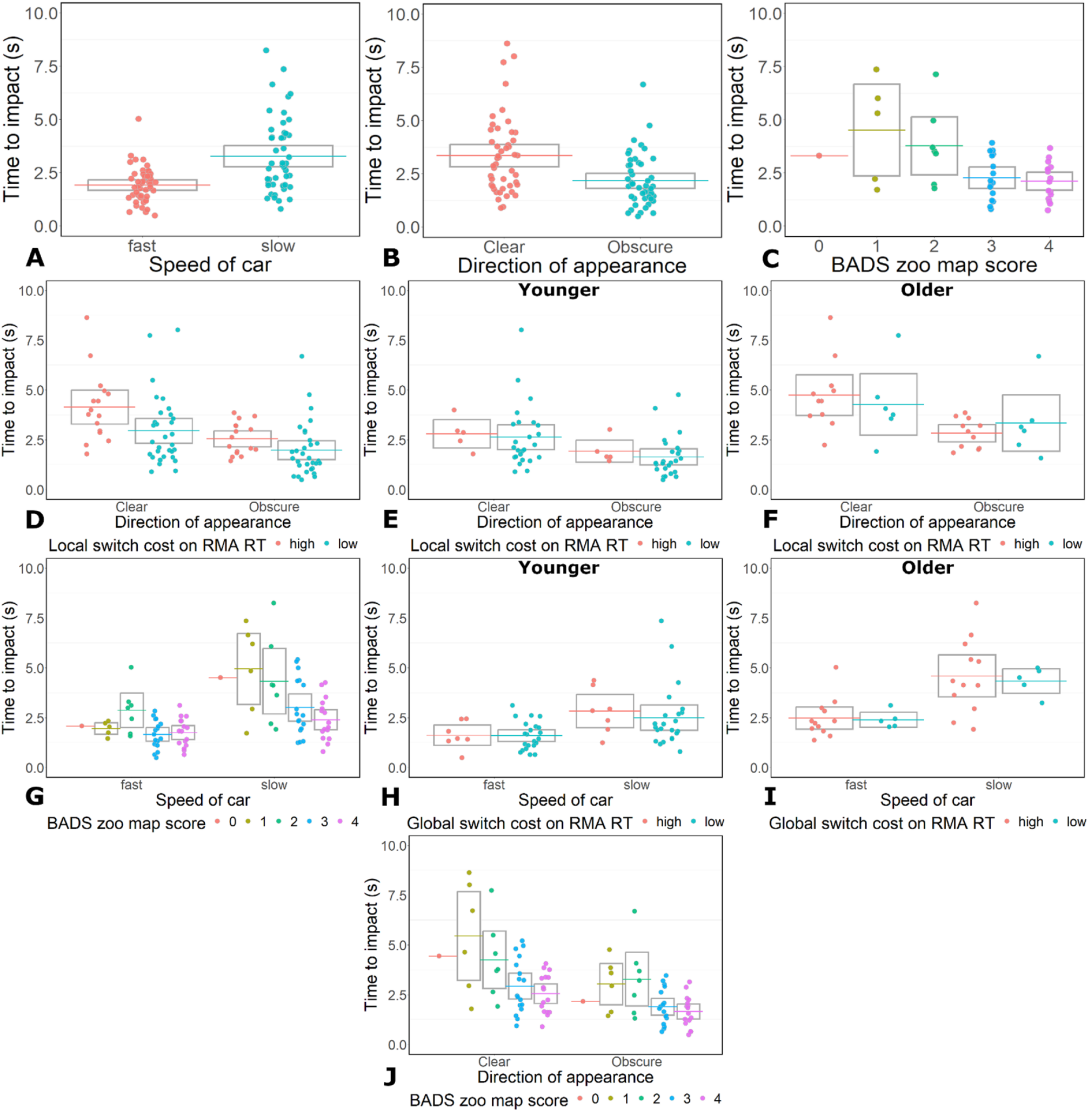
TTI results for Experiment 1. (**A**) Main effect of speed. (**B**) Main effect of car view. (**C**) Main effect of BADS score. (**D**) Interaction between view and local switch costs on the RMA task. Interaction between view and local switch costs on the RMA task for YAs (**E**) and OAs (**F**) as part of the three-way interaction between age, local switch costs, and car view. (**G**) Interaction between BADS score and car speed. (**H**) Interaction between car speed and global switch costs on the RMA task for YAs as part of the three-way interaction between age, global switch costs and car speed. (**I**) Interaction between car speed and global switch costs on the RMA task for OAs as part of the three-way interaction between age, global switch costs and car speed. (**J**) Interaction between BADS score and car view.

There were main effects of car speed (Speed: β = − 1.80, SE = 0.70, t = − 2.58, p = 0.010; Table 2, Fig. 2A). All participants had shorter TTI when cars travelled faster compared to slower (Fig. 2A, Table 2). This reduction in TTI was larger for participants with lower BADS zoo map scores than participants with higher BADS zoo map scores (Fig. 2G; Supplementary Table S2). There was also a three-way interaction between car speed, age group, and global switch costs on RMA RT (Table 2). We ran simple effects LMMs to determine the directions of the three-way interaction (Supplementary Table S2). These indicated that all participants reduced their TTI when cars travelled quickly compared to slowly, and that this reduction was largest for OAs with large global switch costs. (Fig. 2H,I; Supplementary Table S2).

There was a main effect of car view on TTI (View: β = − 2.09, SE = 0.68, t = − 3.07, p = 0.002; Table 2 and Fig. 2B). All participants decreased their TTI when cars travelled from an obscured view compared to a clear view (Fig. 2B, Table 2). This decrease was greater for participants with larger local switch costs than participants with smaller local switch costs on the RMA task, and participants with lower BADS scores compared to higher BADS scores (Fig. 2D,J respectively; Table 2). There was also a three-way interaction between car view, age group, and local switch costs TTI (Fig. 2E,F, Table 2). This three-way interaction also indicated all participants reduced their TTI when cars travelled from an obscured view compared to a clear view, and that this reduction was largest for OAs with large local switch costs (Fig. 2E,F; Supplementary Table S2). The three-way interaction indicated that amongst the YAs the reduction was largest for participants with smaller local switch costs (Fig. 2F, Supplementary Table S2).

There was a main effect of BADS scores on TTI (Table 2, Fig. 2C). Participants with high BADS scores, left less TTI than participants with low BADS scores (Table 2, Fig. 2C).

## Discussion Experiment 1

In Experiment 1 we manipulated the lane the cars travelled along (near/far), the number of lanes the cars travelled along, the speed of the cars, and the view of the cars (clear/obscure). Experiment 1 allowed us to resolve two potential confounds between the number of lanes and the number of traffic directions in Experiment 2.

We found no impact of cars travelling on the near vs the far lane or cars travelling along both lanes vs one lane on the crossing decisions made by participants. Therefore, any effects of the number of directions cars travel from (both vs one) on TTI in Experiment 2 will be due to the travel direction and not the number of lanes, resolving this confound in regards to crossing decisions.

Here we found that OAs did not leave significantly more or less TTI than YAs. As OAs in this sample also did not have significantly different walking speeds than YAs we suggest that the crossing decisions OAs are making are as safe as the crossing decisions YAs are making.

Overall participants with reduced EFs (spatial planning) left more TTI than participants with better EFs. This suggests that despite poorer EFs participants are able to make safe crossing decisions, at least in simple situations.

When participants had less time to make a crossing decision (fast cars or obscured view), they left less TTI compared to when they had more time. Participants with poorer attention switching abilities (local switch costs for obscured view, global for fast cars) reduced their TTI by more than participants with better attention switching abilities. When the view of the car was obscured the reduction in TTI was greatest for OAs with large local switch costs suggesting that participants with poorer attention switching abilities, and in particular OAs with poorer attention switching abilities make risky crossing decisions in this situation. Attention switching involves the process of disengaging with a previously attended task or stimuli, updating WM with a new task or stimuli, and inhibition of distracting stimuli^27,28^. Previous studies have shown that updating WM after a delay leads to poorer performance on the attention switching task than with no delay^29^. In the case of an obscured view there was a delay in seeing the car compared to when the view was clear. Therefore, participants with poorer attention switching abilities may make less safe crossing decisions in the obscured view condition because they take longer to update their WM with new information about car position giving them less time to make a crossing decision. In the case of fast cars, participants with poorer attention switching abilities may not be able to update their WM with the car speed and position, in time to make a safe crossing decision. Compounding this, OAs have slower visual processing speeds than YAs^10–13^, so OAs with poorer attention switching might they have difficulties updating their WM with the new car positions but they are also slower to process all the information needed to make a safe crossing decision. This may explain why OAs with poorer attention switching in particular make riskier crossing decisions when there is less time to make a crossing decision.

We also found that participants with poorer spatial planning abilities reduced their TTI by more than participants with greater spatial planning abilities when they had less time to make a crossing decision (fast cars or obscured cars). This suggests that participants with poorer spatial planning abilities made less safe decisions than participants with better spatial planning abilities when they had less time to make a crossing decision. This may be due to participants with poorer spatial planning abilities being less efficient at executing a plan in a road crossing situation than participants with better spatial planning abilities^25,26^.

### Summary Experiment 1

We found that OAs were able to make safe crossing decisions as they leave the same amount of TTI as YAs, and have the same walking speeds as YAs. In specific situations, such as when participants had little time to make a crossing decision, OAs with poorer EFs and participants with poorer spatial planning abilities begin to have difficulties in making safe crossing decisions as they leave less TTI thus making less safe crossing decisions.

## Introduction Experiment 2

In Experiment 1 we found that in situations where high processing speeds were required OAs with poorer EFs and participants with poorer spatial planning abilities had difficulties making safe crossing decisions. Other situations that might lead to difficulties in making safe crossing decisions include high WM load situations such as when cars travel from multiple directions, when traffic density is high, or distractors are present. In Experiment 2 we examined the impact of these factors, as well as car speed, and cars travelling from an obscured view to determine if high WM load or short processing time conditions impacted OAs abilities to make safe crossing decisions.

## Results Experiment 2

**Figure 3:**
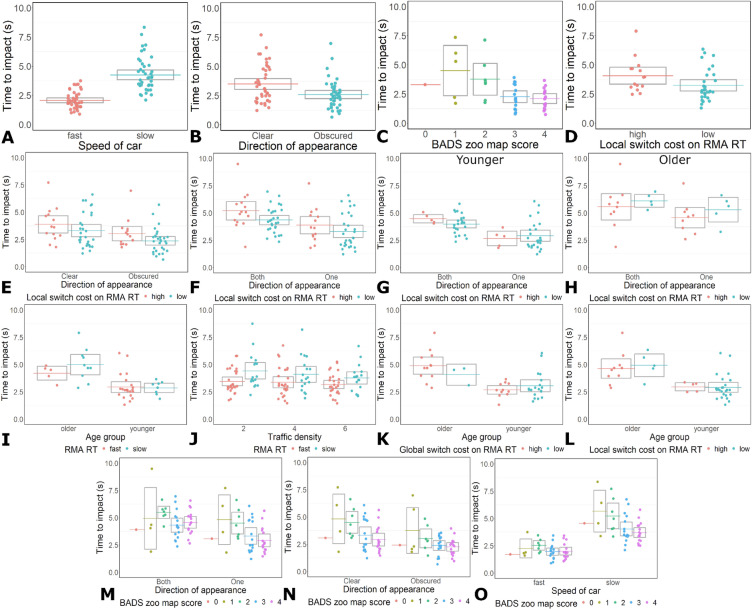
TTI results for Experiment 2. (**A**). Main effect of car speed on TTI. (**B**) Main effect of car view on TTI. (**C**) Main effect of BADS score on TTI. (**D**) Main effect of RMA RT local switch cost on TTI. (**E**) Interaction between cars coming from an obscured view and RMA RT local switch costs on TTI. (**F**) Interaction between cars coming from both directions and local switch cost on TTI. (**G**) Interaction between cars coming from both directions and local switch cost on TTI for YAs, as part of the three-way interaction between age, local switch costs, and cars coming from an obscured view. (**H**) Interaction between cars coming from both directions and local switch cost on TTI for OAs, as part of the three-way interaction between age, local switch costs, and cars coming from an obscured view. (**I**) Interaction between RMA RT and age group on TTI. (**J**) Interaction between RMA RT and traffic density on TTI. (**K**) Interaction between age group and RMA RT global switch costs on TTI. (**L**) Interaction between age group and RMA RT local switch costs on TTI. (**M**) Interaction between BADS score and cars travelling from both directions on TTI. (**N**) Interaction between BADS score and cars travelling from an obscured view on TTI. (**O**) Interaction between BADS score and car speed on TTI.

There was a main effect of car speed on TTI (β = − 3.41, SE = 0.84, t = − 4.07, p = 0.0003, Table 3, Fig. 3A). All participants had shorter TTI when cars travelled quickly compared to slowly. The decrease in TTI was larger for participants with lower BADS zoo map scores than participants with higher BADS zoo map scores (Fig. 3O, Supplementary Table S4).

**Table 3:** LMM results for the TTI left by participants in Experiment 2.

Factor	β	SE	t-value	p-value
Car speed	− 3.41	0.84	− 4.07	0.0003
Car speed * BADS	0.52	0.16	3.24	0.003
Both directions * BADS	0.36	0.16	2.27	0.030
Both directions * Local switch costs	5.10	2.93	3.02	0.005
Both directions * Age * Local switch cost	− 3.71	1.81	− 2.05	0.049
Car view	− 2.89	0.79	− 3.68	0.0001
Car view * Local switch costs	4.06	1.69	2.41	0.022
Car view * BADS	0.42	0.15	2.76	0.010
BADS	− 0.90	0.28	− 3.27	0.002
Local switch cost	− 8.79	3.07	− 2.86	0.007
Local switch cost * Age	8.60	3.29	2.62	0.012
Global switch cost * Age	0.56	0.26	2.20	0.038
RMA RT * Age	− 7.60	2.92	− 2.61	0.015
RMA RT * Traffic density	− 0.12	0.06	− 2.02	0.043

Only significant results are listed. For full LMM results see Table 3 in the Supplementary Materials. See the statistical analysis subsection of the Methods for the models that were run.

The LMMs showed a main effect of car view on TTI (β = − 2.89, SE = 0.79, t = − 3.68, p = 0.0001, Table 3, Fig. 3B). All participants decreased their TTI when cars travelled from an obscured view compared to a clear view. The decrease in TTI was greater for participants with lower BADS scores than participants with higher BADS scores, and for participants with smaller local switch costs than participants with larger local switch costs on the RMA task (Fig. 3N,E, Supplementary Table S4).

The LMM on TTI showed an interaction between spatial planning ability and car travel direction, and between attention switching ability and car travel direction (BADS: β = 0.42, SE = 0.15, t = 2.76, p = 0.010; attention switching: β = 4.06, SE = 1.69, t = 2.41, p = 0.022, Table 3, Fig. 3F,M). All participants had longer TTI when cars travelled in both directions compared to just one direction. The differences were greater for participants with higher BADS zoo map scores than participants with lower scores, and for participants with larger local switch costs than participants with smaller local switch costs on the RMA task (Fig. 3F,M, Supplementary Table S4). There was a three-way interaction between age group, car travel direction, and local switch costs on TTI (Fig. 3G,H; Table 3). Examining the simple effects associated with this three-way interaction revealed that all participants increased their TTI when cars travelled from both directions and this increase was amplified for participants with large local switch costs, and YAs with large local switch costs had the greatest increase in TTI. (Fig. 3G,H; Supplementary Table S4).

The LMM also showed a main effect of local switch costs and BADS on TTI (Local switch costs: β = − 8.79, SE = 3.07, t = − 2.86, p = 0.007; BADS: β = − 0.90, SE = 0.28, t = − 3.27, p = 0.002. Table 3, Fig. 3D,C). Participants with larger switch costs and participants with low BADS scores left more TTI than participants with smaller switch costs and participants with high BADS scores (Supplementary Table S4). There was also an interaction between switch costs (global and local) and age group (Table 3, Fig. 3K,L); and between age and RMA RT (Table 3, Fig. 3I). However, for each of these interactions the impact of switch costs, and RMA RT on YAs and OAs was not significant (Supplementary Table S4).

There was also an interaction between RTs on the RMA test and traffic density on TTI (β = − 0.12, SE = 0.06, t = − 2.02, p = 0.0443, Fig. 3J, Table 3). All participants decreased their TTI with increasing traffic density. This decrease was amplified for participants with slow RTs on the RMA task compared to participants with fast RTs on the RMA task (Fig. 3J, Supplementary Table S4).

## Discussion Experiment 2

In Experiment 2 we examined how the complexity of road crossing situations impacted crossing decisions made by OAs and YAs with varying levels of EFs. We manipulated the complexity of the road crossing situation by varying car speed, the direction the cars travelled in (both or one direction), the initial viewpoint of the cars (clear vs obscure), the traffic density, and the presence of pedestrian distractors.

Overall, and in a replication of Experiment 1, we found that when participants were given less time to make a crossing decision (fast cars or obscured view) they leave less TTI than when they were given more time to make a crossing decision, and that these effects were amplified for participants with poorer EFs (spatial planning and attention switching).We also replicated the finding that OAs did not significantly differ from YAs in the amount of TTI that they left. We discussed these results in the discussion section of Experiment 1.

An interaction between RTs on the RMA task and traffic density was found. Participants with slower RTs on the RMA task decreased their TTI by more than participants with faster RTs when traffic density increased. It is not initially clear why participants that have slower RTs would have difficulties on a more difficult spatial task rather than a task that requires faster response e.g. cars travelling faster. One explanation could be that participants with slower RTs may have slower motor functions in general, so they may be slower to initiate saccades, preventing them from taking in information from each of the cars in time to make a safe crossing decision. However, without eye tracking data it is not possible to determine this with our current data. Future research involving VR and eye tracking would allow for this to be confirmed.

Some road crossing situations were handled well by all participants. For example, when cars travelled from both directions compared to one direction participants increased the amount of TTI they left. This suggests that participants were able to identify that this was a more dangerous situation and they approached the road crossing more cautiously. This was particularly the case for participants with better spatial planning scores compared to participants with poorer spatial planning scores. Spatial planning involves anticipating where objects will appear along a route^30^. Therefore, they can better anticipate the location of the cars coming from the direction they are not attending to, allowing them to make safe crossing decisions.

Participants with poorer attention switching (local switch costs) also increased their TTI by more than participants with better attention switching abilities when cars travelled from both directions compared to one direction. This was particularly the case for YAs with larger switch costs as had the largest increase in TTI. Perhaps YAs faster visual processing speed allowed them to compensate for their larger attention switching costs, and still process the necessary information to make a crossing decision earlier than OAs. Alternatively, participants with poorer attention switching have an awareness of their reduced abilities and so they are extra cautious in more difficult road crossing situations. However, the reason for these results are not clear and would need further investigation to determine the explanation for these effects.

There were interactions between age and attention switching, between age and RTs on the RMA task, on the amount of TTI participants left. However, none of the simple effects models used to examine the direction of the interaction were significant, therefore, we consider these results to not be robust enough to discuss further.

## General discussion

In these two experiments we investigated whether OAs had difficulties making crossing decisions in specific road crossing situations or whether OAs had general difficulties making crossing decisions in all situations. We examined this by presenting participants with virtual road crossing scenarios of varying complexity. Complexity was manipulated along two main themes, increasing cognitive load, and reducing the time participants had to make a crossing decision. Overall, we found that OAs were able to make safe crossing decisions, as they made similar crossing decisions and walking speeds to YAs. This was the case even in high cognitive load situations such as cars travelling from both directions, or pedestrian distractors being present. However, in situations where participants had less time to make a crossing decision all participants had difficulties making safe crossing decisions which was particularly apparent for OAs and participants with poorer EFs.

All participants made less safe crossing decisions when they had less time to make crossing decisions (fast cars or obscure view) than when they had more time. This may point towards cognitive processing speed limits being reached. The limits could be on the bottom-up end or the top-down end, or both. If there is not enough time to perceive the required perceptual information (bottom-up processing limits are reached) in the situation, then there will be less information passed forward to decision making processes, and crossing decisions will need to be made with less information than in situations where participants have more time. Alternatively, participants may still be able to perceive all the required information to make a crossing decision but they may not be able to go through all the decision-making processes required to make a safe crossing decision or initiate a crossing movement (top-down limits are reached). Further research is needed to tease apart whether top-down or bottom-up processing limits are reached.

Our finding that YAs and OAs made less safe crossing decisions when cars travelled quickly has implications for infrastructure development of roads around crossing locations, residencies, and cities. At their fastest the cars travelled at 70 km/h which is not an uncommon speed for vehicles to travel at even in cities and residential areas, but already participants of all ages were making less safe crossing decisions. We suggest that larger areas in cities should have speed limits, and in particular, around care homes or retirement villages where there are currently no formal recommendations to have speed limits in places as for schools^31^.

All participants had difficulties in making safe crossing decisions when the view of the oncoming cars was initially obscured. This has implications for infrastructure around road crossing locations. In the real world there are often cars parked near crossing areas or trees with overhanging branches blocking the view of oncoming traffic at crossing locations. We suggest that limits for how close cars can park to the crossing should be lengthened and that trees around crossing locations are managed such that they do not block the view of oncoming vehicles.

There were situations where all participants performed well. In high cognitive load situations participants continued to make safe crossing decisions. For example, when cars travelled from both directions all participants were more cautious compared to when cars travelled from one direction. Moreover, when pedestrian distractors were present and when traffic density was high participants did not change their behaviour suggesting they were able to ignore distractors and take into account high traffic densities, possibly by visually grouping the cars^32,33^.

### Limitations

Our VR environment was a 180° desktop setup rather than a head-mounted device or a CAVE with motion tracking. With the desktop setup we were unable to examine how having to perform a motor task in combination with an increasingly difficult perceptual decision making task (road crossing) would impact on crossing performance. Indeed previous studies have shown making crossing decisions with walking, or with another task leads to less safe crossing decisions being taken among OAs^17,18^.

Furthermore, our OA sample was limited in size due to the COVID-19 pandemic. Therefore, the power of the current study is likely lower than with a larger sample size.

## Conclusions

Overall, we found that observers make less safe crossing decisions when they have less time to make a crossing decision (i.e. high speed, visual occlusion). This is especially the case for OAswith poorer EFs. Our findings have important implications for road crossing infrastructure and policy. Our data suggests that more effort should be taken to make sure that all road crossing points are clear of visual obstructions to ensure that participants have as much time to make a crossing decision as possible. Furthermore, we suggest more speed limits should be placed around retirement or care homes which is currently not legislated for in the UK and Australia.

## Methods

### Participants

Fifty-three participants were recruited, 19 aged between 65 and 85 years old (y/o, mean = 70.80, SE = 1.31, range 65–85 y/o), and 34 aged between 18 and 24 y/o (mean = 19.94, SE = 0.26, range 18–24 y/o). All YAs were recruited from Bournemouth University, UK. All participants took part in both experiments in the same testing session. Older adults were recruited either from the Bournemouth Ageing and Dementia Research Centre (ADRC) participant pool or from the Wimborne branch of the University of the Third Age. All participants had normal or corrected to normal vision. Participants were screened for mild cognitive impairment using the Montreal Cognitive Assessment (MoCA,^34^). No participants scored below the cut off score of 23^35^. Therefore, all participants were included in the final analyses. The study was approved by the Bournemouth University ethics committee. Informed consent was obtained from participants prior to taking part. Participants took part in exchange for course credits or monetary compensation for their time. This study was performed in accordance with all appropriate institutional guidelines and international guidelines and regulations, in line with the principles of the Helsinki Declaration.

### Executive function tests

EF abilities were assessed using the BADS zoo map test^36^, and the Rogers and Monsell^37^ attention shift paradigm (RMA).

The BADS zoo map test assessed the participants’ spatial planning ability by assessing participants’ ability to plan a route around a zoo. In the first trial participants were given a map of a zoo and instructed to plan a route around a zoo, starting at the entrance and finishing with a picnic. Along the route participants had to visit specified locations in any order while they followed set rules, such as only using specified paths twice and not visiting unspecified locations. Participants’ planning time and time to complete the task was recorded. In the second trial participants had to plan a route around the same zoo, followed the same rules, and visited the same locations but in a specified order. Again, the participants’ planning time and time to complete the task was recorded. Participants’ performance was assessed based on visiting the correct locations and points were deducted when participants broke the rules and exceeded time limits for planning on the second trial. The scores ranged from zero to four, the higher the score the better participants performed on the test.

The RMA assesses participants’ attentional control by instructing participants to switch between two similar tasks. Participants were presented with number letter pairs (e.g., 9E) and depending on the position of the stimulus on the screen they either had to identify whether the number was odd or even or whether the letter was a vowel or consonant. For the RMA task I extracted the global and local switch costs as done by Rogers and Monsell^37^. The global switch costs refer to the difference in performance between a block where participants perform the same task and a block where participants are switching between tasks. Local switch costs refer to the differences in performance between switch and non-switch trials. I also extracted the participants’ accuracy and response times on each trial of the RMA. Correct responses were scored as one, incorrect responses as zero. Individual performance was then assessed by averaging accuracy over the entire RMA experiment.

### Walking speed

Participants’ walking speed was measured by asking participants to walk along a nine-metre corridor while measuring their walking time. Participants were asked to walk at their normal day to day walking pace. This was done three times and an average walking time was then calculated.

### Apparatus

For both experiments stimuli were presented across three Samsung monitors, each with a screen resolution of 1920 by 1080 pixels, an aspect ratio of 16:9, a width of 88.6 cm, a height of 49.8 cm, and a refresh rate of 100 Hz. The left and right screens were placed at 60° angle to the centre screen. Participants were seated at a distance of 100 cm (setup shown in Fig. 6A). The screens had a combined horizontal viewing angle of 180° and a vertical viewing angle of 32°. The experiment was coded in Worldviz Vizard 5.0 using Python 2.7 and the PyLink Toolbox extensions^38^. Head position and orientation were recorded using the Polhemus Fastrak motion tracking system with a sampling rate of 120 Hz.

### Procedure

Both experiments used a virtual road crossing environment created in 3DS Max and Maya (Fig. 6B,C) which was made to simulate the road crossing scene used in^39^. At the beginning of the experiment participants were informed that they would be presented with a series of road crossing situations on screen and that they would have to indicate by pressing the spacebar on a keyboard when they could cross the road and hold the key pressed for as long as they thought it was safe to cross. At the start of each experimental block participants were informed about which side the cars would appear from—left hand side, right hand side, or both sides (Experiment 2 only). Vehicles travelled at two speeds—249 (slow) or 583 (fast) virtual world units per second. This was equivalent to approximately 30 and 70 km/h respectively. Each trial started with the presentation of a central fixation cross. Once the participants had fixated on the cross, the virtual environment was presented. Each trial was followed by a black screen with text stating the trial had ended and the participant should press the spacebar to continue. Immediately after the participants pressed the spacebar the next trial would start with the central fixation cross.

### Design Experiment 1

**Figure 4:**
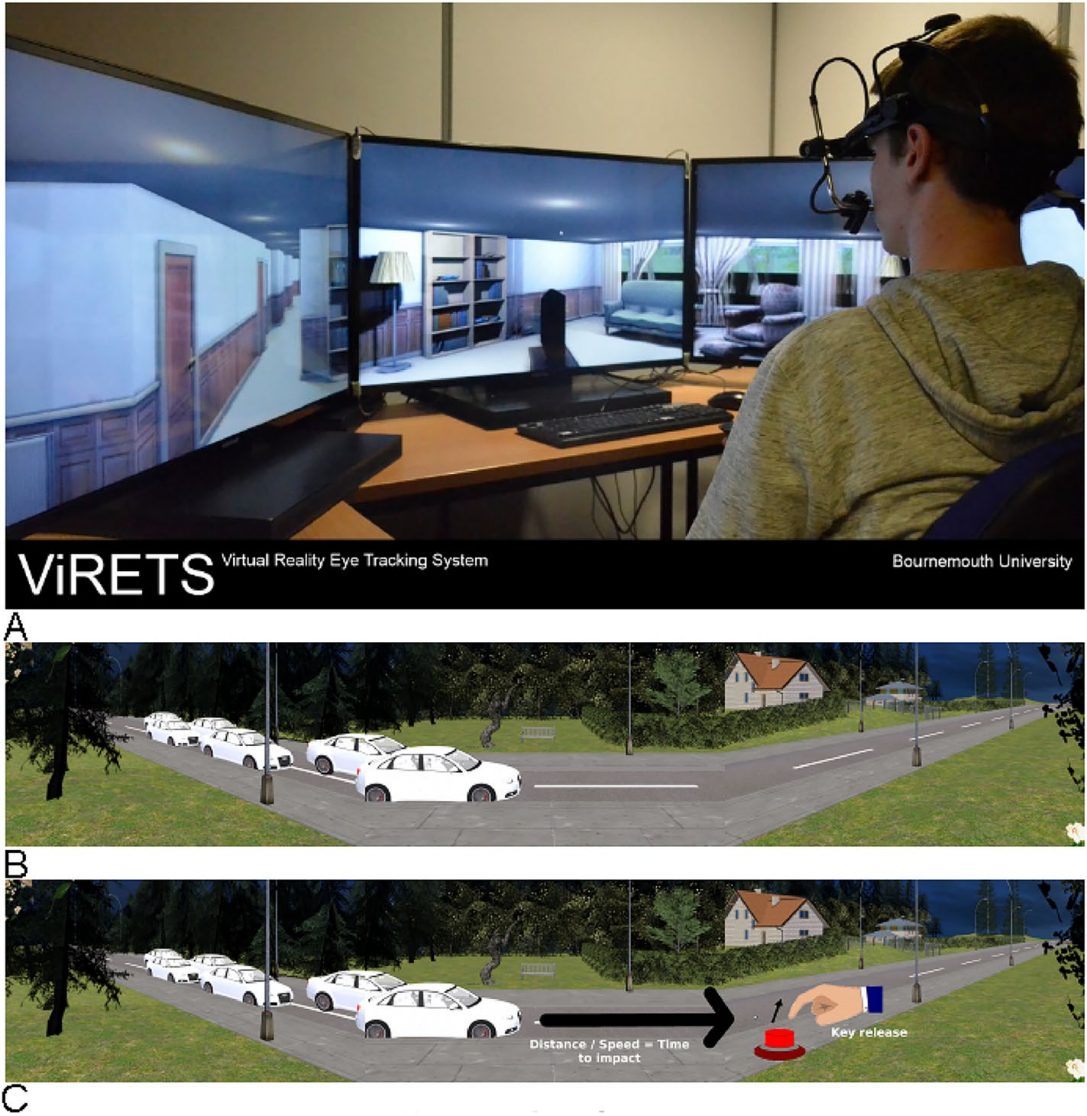
(**A**) An example of the virtual reality setup in the lab with a lab member wearing the Eyelink II eye tracker and a head motion tracker fixed to the eye tracker. We tracked participants’ head movements but did not track their eye movements for this experiment. (**B**) Screenshot of the stimulus presented to the participant. The stimulus was split across the three screens in (**C**). Cars could travel from left to right or from right to left. (**c**) Visual description of the time to impact measure. Participants would press a key on the keyboard when they thought it was safe to cross, and hold the button down until they felt it was no longer safe to cross. When participants released the key, we would calculate the time it would take the vehicle to reach the participants position in the virtual environment. This would form our time to impact measure. For more details on the time to impact measure see the Road crossing task subsection. (**A**) was an image taken by one of the authors of the VR lab setup. (**B**) and (**C**) were screenshots taken of the virtual environment, the annotations in (**C**) created using Gnu Image Manipulation Program (GIMP) v.2.10.34 (The GIMP Development Team. (2019). GIMP. Retrieved from https://www.gimp.org).

**Figure 5:**
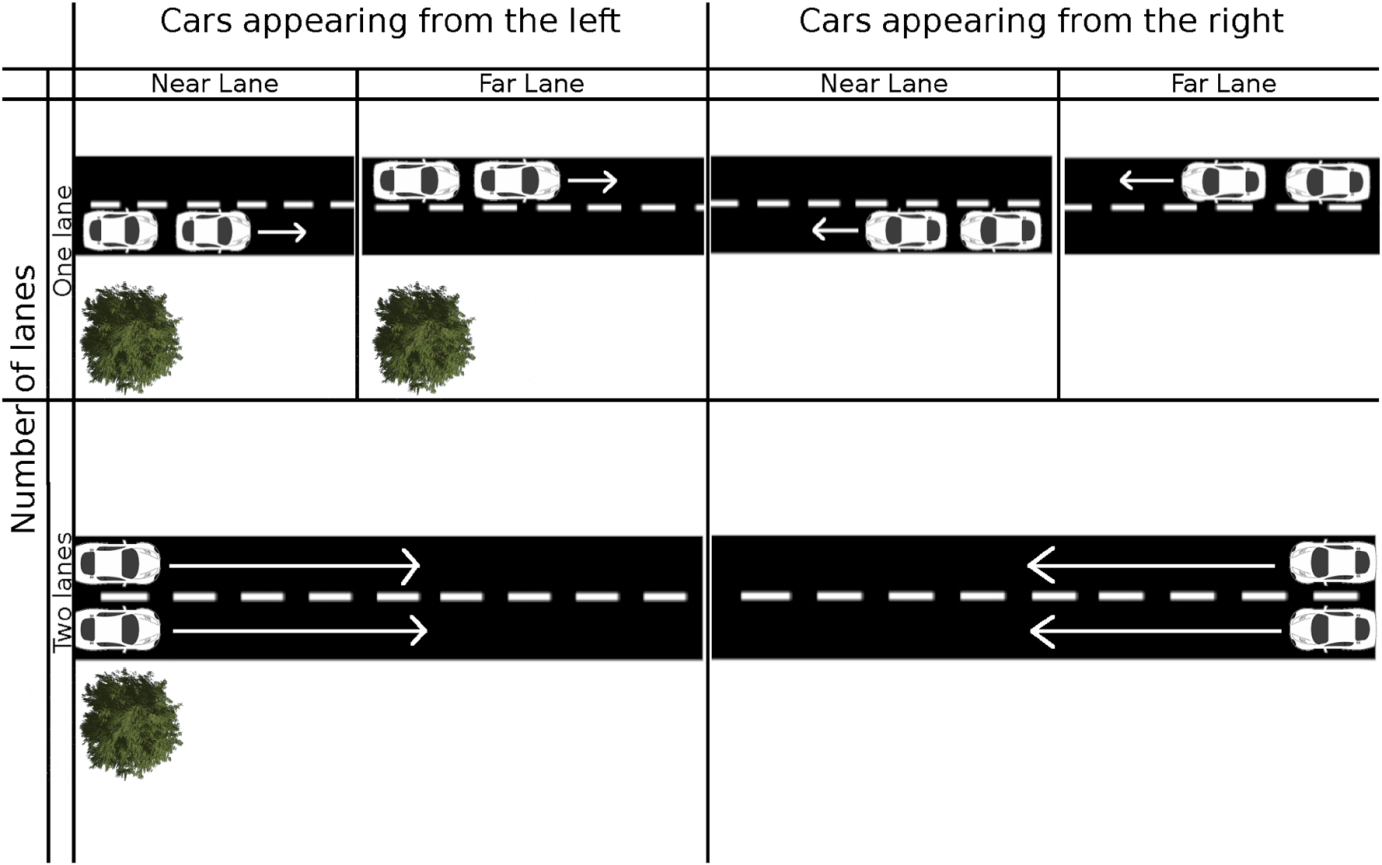
A schematic for the conditions manipulated in Experiment 1. The trees indicate the trials where the views of the cars were obscured. The rows of the schematic indicated the number of lanes the cars travelled along. The columns indicate whether the cars travelled along the lane closest (near) or furthest (far) from the participants (when cars travelled in one lane only).

In this experiment 30 trials were presented to participants, split into two blocks of 15 trials. Each trial lasted 15 s. For one block the cars travelled from left to right, and on the other cars travelled from right to left. The view of the cars that travelled from left to right were occluded by trees (Fig. 4B,C). The view of the cars that travelled from right to left was not obstructed. On each trial two cars were presented. For half the trials both cars travelled along one lane, either the near or the far lane. For the other half of the trials the cars travelled in both lanes but in the same direction. Four different car models were presented randomly—Audi S4, Toyota Prius, Volkswagen Polo, and Volkswagen Beetle. All car models were coloured white, except for the Polo that was coloured red. All cars in a given trial were of the same model. The speed of the cars was randomly set to either 30 or 70 km/h but all cars presented on a given trial moved at the same speed. A summary of the conditions is presented in Fig. 5 with the exception of car speed and car model.

### Design Experiment 2

**Figure 6:**
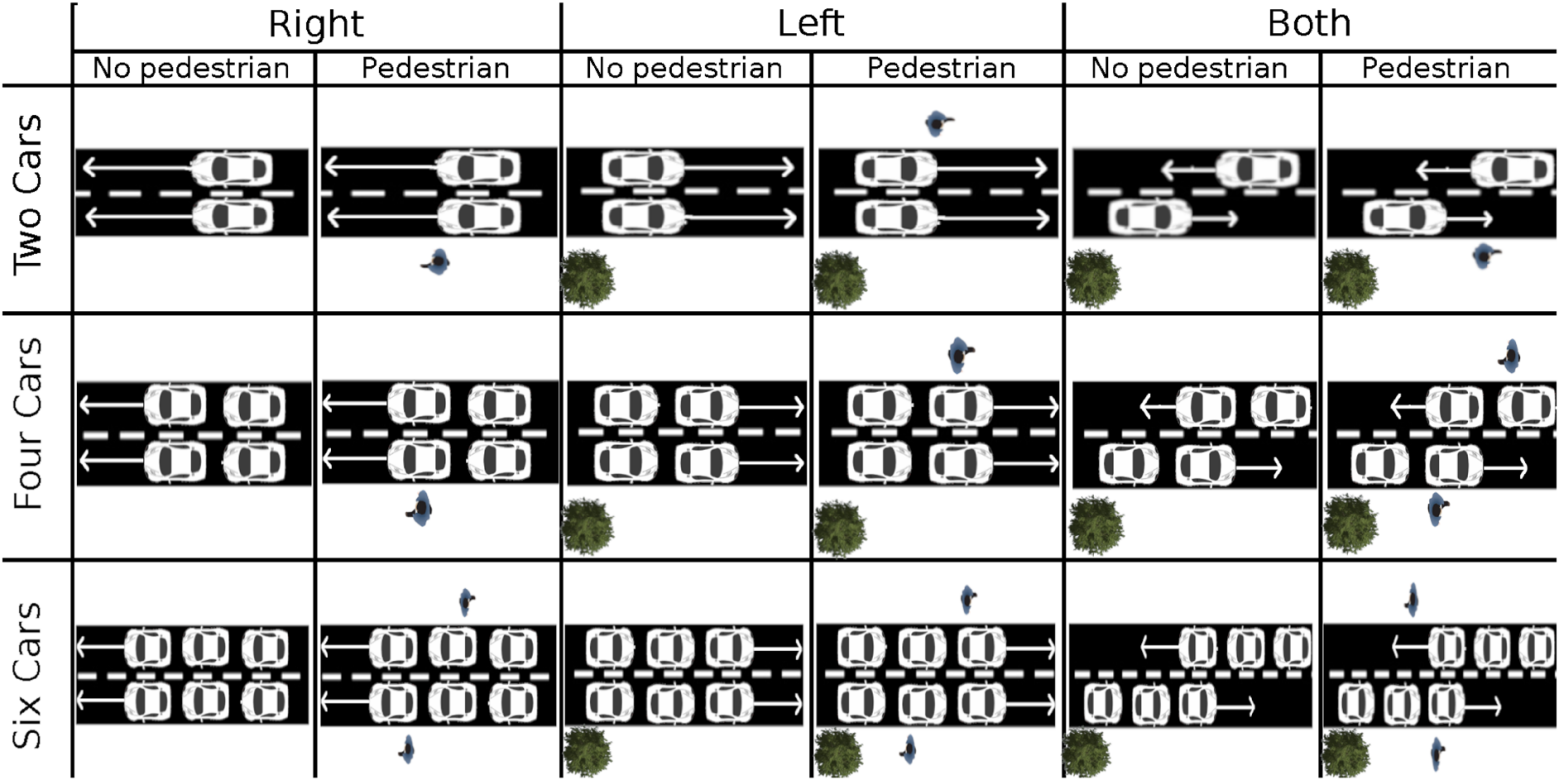
Conditions for Experiment 2. Task complexity was manipulated by varying traffic density, direction, pedestrian distractors and occlusions. Trees in the third to the sixth column indicate trials where the views of the cars were initially obscured. The number of cars were manipulated at three levels starting from two (top row), four (middle) and higher complexity condition of six (bottom row).

In this experiment 120 trials were presented to participants, split into three blocks of 40 trials, each trial lasted for 15 s. On each trial cars travelled along both lanes, the car travel direction was different for each block. In the three blocks cars travelled either from left to right, from right to left, or in both directions. The view of the cars that travelled from left to right was slightly obscured by trees. The view of the cars that travelled from right to left was not obstructed. The number of cars presented on each trial varied between two, four, and six cars. On half the trials in each block the car speed was fast (70 km/h) and on the other half the car speed was slow (30 km/h). All cars presented in a trial travelled at the same speed. In half the trials in each block pedestrian avatars (i.e. distractors) were present that walked along the near or far sidewalk or stood still. The number of pedestrians presented on the trials varied randomly between one and two pedestrians. The same four car models as in Experiment 1 were used in this experiment and were also randomly varied. A summary of the conditions is presented in Fig. 6 with the exception of car speed.

### Road crossing task

For the task participants watched the road crossing videos described in the Procedure subsection and Fig. 4. While they watched the videos, they were instructed to indicate by pressing the spacebar on a keyboard when they could cross the road and hold the key pressed for as long as they thought it was safe to cross. By asking participants to hold the key down it would be a proxy for the duration participants would take to cross the road.

From these crossing decisions we calculate a “Time to impact” (TTI) measure, which is the main dependent variable used to assess the safety of crossing decisions. We define TTI as the time that it would take for the closest approaching vehicle, in each lane, to reach the participants, from the moment when the participants stopped indicating that crossing was safe (i.e. when they released the spacebar indicating that it was no longer safe to cross). This is illustrated in Fig. 4C. When the TTI is long then the cars are far away from the virtual position participants are at in the virtual environment. When the TTI is short the cars are close to the virtual position the participants are in the virtual environment. We consider longer TTIs to be safer than shorter TTIs.

### Statistical analyses

All statistical analyses and figures were created and performed using Matlab^40^ and R version 3.6.3^41^. In all analyses age is examined as a categorical variable, YAs aged between 18 and 24 y/o, and OAs aged between 65 and 85 y/o. We trimmed the distributions by 10% before running our analyses to reduce the influence of outliers in our data.

#### Crossing decisions

TTI in both experiments was analysed with linear mixed models (LMMs). In Experiment 1 the model included fixed effects of age group (above or below 60y/o), number of lanes, near or far lane, car speed, car direction, direction of travel (from the left or right), RMA RTs, zoo map score, global switch cost on RMA RTs, local switch cost on RMA RTs. The model included interactions between age, each of the EF measures, and each of the task conditions. The model also included random intercepts for each participant and each trial. To begin with, the model contained random slopes for each fixed factor but the model did not converge so all random slopes were removed. All the models that were run are listed in Supplementary Table S5. The full model is described in Eq. 1.

1 $$\begin{aligned} & {\text{TTI }}\sim {\text{RMA RT }}* \, \left( {{\text{Age }}*{\text{ Speed }} + {\text{ Age }}*{\text{ View }} + {\text{ Age }}*{\text{ nLanes}} + {\text{ Age }}*{\text{ Near}}/{\text{Far Lane}}} \right) \\ & \quad + {\text{ local }}* \, \left( {{\text{Age }}*{\text{ Speed }} + {\text{ Age }}*{\text{ View }} + {\text{ Age }}*{\text{ nLanes}} + {\text{ Age }}*{\text{ Near}}/{\text{Far Lane}}} \right) \, \\ & \quad + {\text{ global }}*\left( {{\text{Age }}*{\text{ Speed }} + {\text{ Age }}*{\text{ View }} + {\text{ Age }}*{\text{ nLanes}} + {\text{ Age }}*{\text{ Near}}/{\text{Far Lane}}} \right) \, \\ & \quad + {\text{ BADS }}* \, \left( {{\text{Age }}*{\text{ Speed }} + {\text{ Age }}*{\text{ View }} + {\text{ Age }}*{\text{ nLanes}} + {\text{ Age }}*{\text{ Near}}/{\text{Far Lane}}} \right) \\ & \quad + \, \left( {{1 }|{\text{ participant}}} \right) \, + \, \left( {{1 }|{\text{ video}}} \right) \\ \end{aligned}$$

In Experiment 2 the model included fixed effects of age group (above or below 60y/o), traffic density, presence of distractors, car speed, direction of travel (from the left, right, or both directions), RMA RTs, zoo map score, global switch cost on RMA RTs, local switch cost on RMA RTs. The model included interactions between age, each of the EF measures, and each of the task conditions. The model also included random intercepts for each participant and each trial, and random slopes for age group, car speed, and direction of travel. To begin with, the model contained random slopes for each fixed factor but the model did not converge so the majority of the random slopes were removed. This model initially included interactions for cars appearing from both directions and car speed, cars appearing from an obscured viewpoint, traffic density, and pedestrian presence. This model did not converge so these interactions were removed. All the models that were run are listed in Supplementary Table S6. The final model is described in Eq. 2.

2 $$\begin{aligned} & {\text{TTI }}\sim {\text{ BADS}}*\left( {{\text{Age }}*{\text{ Direction }} + {\text{ Age }}*{\text{ Speed }} + {\text{ Age }}*{\text{ Traffic }} + {\text{ Age }}*{\text{ Pedestrians}}} \right) \\ & \quad + {\text{Global}}*\left( {{\text{Age }}*{\text{Direction }} + {\text{ Age }}*{\text{ Speed }} + {\text{ Age }}*{\text{ Traffic }} + {\text{ Age }}*{\text{ Pedestrians}}} \right) \\ & \quad + {\text{Local}}*\left( {{\text{Age }}*{\text{Direction }} + {\text{ Age }}*{\text{ Speed }} + {\text{ Age }}*{\text{ Traffic }} + {\text{ Age }}*{\text{ Pedestrians}}} \right) \\ & \quad + {\text{RMA RT}}*\left( {{\text{Age }}*{\text{ Direction }} + {\text{ Age }}*{\text{ Speed }} + {\text{ Age }}*{\text{ Traffic }} + {\text{ Age }}*{\text{ Pedestrians}}} \right) \\ & \quad + \left( {{1 } + {\text{ Age }} + {\text{ Speed }} + {\text{ Direction }}|{\text{ participant}}} \right) \\ & \quad + \left( {{1 } + {\text{ Age }} + {\text{ Speed }} + {\text{ Direction }}|{\text{video}}} \right) \\ \end{aligned}$$

All significant interactions were investigated using simple effects LMMs with a Tukey HSD correction for multiple comparisons. Interactions that involved EF measures were examined by splitting the sample (OAs and YAs combined) EF data with a mean split to create “higher” and “lower” EF groups. The simple effects LMMs were then performed on the higher and lower EF groups individually.

#### Executive function tests

Differences between older and younger adults on all executive functioning measures were determined using a bootstrap t-test with 20% trimmed means. Multiple comparisons were corrected using the Hochberg method. Bootstrap t-tests were used as they handle skewed distributions and outliers better than the Student’s t-test^42^. Bayes factors were also calculated using the BayesFactor package in R^43^, after outliers were removed using the median absolute deviation (MAD) rule.

### Supplementary Information

Supplementary Information.

## Data Availability

All data, and analysis scripts are openly available at: https://doi.org/10.18746/bmth.data.00000171.
