# Supplementary material for: The impact of perceptual complexity on road crossing decisions in younger and older adults
Source: Sci Rep. 2024 Jan 4;14:479. doi: 10.1038/s41598-023-49456-9 (PMC10767050; doi:10.1038/s41598-023-49456-9)
Supplement: Supplementary file 1 — Supplementary Information. [file 41598_2023_49456_MOESM1_ESM.docx]

**Supplementary Materials**

Experiment 1

Supplementary Table S1. Full LMM results for the TTI judgements made by participants on Experiment 1. Significant results are highlighted in blue. See the Methods section for the model that was run.

| *Factor* | *β* | *SE* | *t-value* | *p-value* |
| --- | --- | --- | --- | --- |
| Car speed | -1.80 | 0.70 | -2.58 | 0.010 |
| Car speed * BADS | 0.41 | 0.13 | 3.06 | 0.002 |
| Car speed * Age * Global switch cost | 2.27 | 0.93 | 2.45 | 0.014 |
| Car speed * Age | 1.86 | 1.38 | 1.34 | 0.180 |
| Car speed * RMA RT | -0.66 | 0.37 | -1.79 | 0.074 |
| Car speed * Local switch cost | 2.03 | 1.44 | 1.41 | 0.159 |
| Car speed * Global switch cost | -0.019 | 0.34 | -0.054 | 0.957 |
| Car speed * Age * RMA RT | -1.77 | 0.97 | -1.82 | 0.069 |
| Car speed * Age * BADS | -0.206 | 0.19 | -1.07 | 0.283 |
| Car speed * Age * Local switch cost | -2.04 | 1.60 | -1.28 | 0.200 |
| View * Age | -0.27 | 1.34 | -0.21 | 0.838 |
| View * RMA RT | -0.35 | 0.37 | -0.97 | 0.334 |
| View * Global switch cost | -0.28 | 0.33 | -0.83 | 0.405 |
| View * Age * RMA RT | 0.34 | 0.93 | 0.36 | 0.719 |
| View * Age * Global switch cost | 0.68 | 0.88 | 0.78 | 0.435 |
| View * Age * BADS | -0.09 | 0.19 | -0.51 | 0.613 |
| BADS | -0.97 | 0.25 | -3.85 | 0.0003 |
| Lane number | -0.03 | 0.83 | -0.03 | 0.974 |
| Lane number * Age | -1.80 | 1.63 | -1.10 | 0.270 |
| Lane number * RMA RT | -0.07 | 0.45 | -0.15 | 0.877 |
| Lane number * Local switch cost | -0.17 | 1.66 | -0.10 | 0.919 |
| Lane number * Global switch cost | 0.11 | 0.41 | 0.28 | 0.784 |
| Lane number * BADS | 0.03 | 0.16 | 0.20 | 0.838 |
| Lane number * Age * RMA RT | 1.49 | 1.13 | 1.32 | 0.188 |
| Lane number * Age * Local  switch cost | 0.21 | 1.81 | 0.12 | 0.907 |
| Lane number * Age * Global switch cost | -0.75 | 1.05 | -0.72 | 0.472 |
| Lane number * Age * BADS | 0.03 | 0.22 | 0.12 | 0.908 |
| Near/Far Lane | -0,57 | 0.81 | -0.71 | 0.480 |
| Near/Far Lane * Age | 2.35 | 1.61 | 1.46 | 0.146 |
| Near/Far Lane * RMA RT | 0.34 | 0.43 | 0.80 | 0.427 |
| Near/Far Lane * Local switch cost | 1.30 | 1.66 | 0.78 | 0.434 |
| Near/Far Lane * Global switch cost | -0.31 | 0.39 | -0.81 | 0.417 |
| Near/Far Lane * Age * BADS | -0.07 | 0.22 | -0.30 | 0.762 |
| RMA RT | -0.13 | 0.75 | -0.18 | 0.859 |
| Age | -1.99 | 2.72 | -0.73 | 0.468 |
| Local switch cost | -4.47 | 2.79 | -1.60 | 0.115 |
| Global switch cost | 0.866 | 0.65 | 1.33 | 0.190 |

Supplementary Table S2. Results of simple effects LMMs run to determine the direction of the interaction from the significant interactions found in Table 1. Simple effects models were run using the following equation: TTI ~ Factor (one level only) + (1|participant) + (1|trial). Values are corrected for multiple comparisons using a Tukey HSD correction.

| *Factor* | *β* | *SE* | *t-value* | *p-value* |
| --- | --- | --- | --- | --- |
| Car speed – low BADS | -1.14 | 0.11 | -10.41 | <1x10^-10^ |
| Car speed – high BADS | -1.10 | 0.09 | -11.77 | <1x10^-10^ |
| Car speed – YAs with small global switch costs | -0.83 | 0.13 | -6.34 | 2.37x10^-10^ |
| Car speed – YAs with large global switch costs | -0.92 | 0.14 | -6.59 | 4.53x10^-11^ |
| Car speed – OAs with small global switch costs | -2.00 | 0.25 | -8.13 | 4.44x10^-16^ |
| Car speed – OAs with large global switch costs | -2.07 | 0.28 | -7.30 | 2.86x10^-13^ |
| View – low BADS | -1.15 | 0.11 | -10.57 | <1x10^-10^ |
| View – high BADS | -1.08 | 0.09 | -11.64 | <1x10^-10^ |
| View – low local switch cost | -0.93 | 0.10 | -9.24 | <1x10^-10^ |
| View – high local switch cost | -1.53 | 0.21 | -7.32 | <1x10^-10^ |
| View – YAs with small local switch costs | -0.92 | 0.11 | -8.31 | <2x10^-16^ |
| View – YAs with large local switch costs | -0.86 | 0.21 | -4.18 | 2.93x10^-5^ |
| View – OAs with small local switch costs | -1.01 | 0.24 | -4.20 | 2.71x10^-5^ |
| View – OAs with large local switch costs | -1.81 | 0.29 | -6.29 | 3.09x10^-10^ |

Experiment 2

Supplementary Table S3. Full LMM results for the TTI judgements made by participants on Experiment 2. Significant results are highlighted in blue. See the Methods section for the model that was run.

| *Factor* | *β* | *SE* | *t-value* | *p-value* |
| --- | --- | --- | --- | --- |
| Car speed | -3.42 | 0.84 | -4.07 | 0.0003 |
| Car speed * Age | -3.13 | 2.31 | -1.36 | 0.183 |
| Car speed * BADS | 0.52 | 0.16 | 3.24 | 0.003 |
| Car speed * Local switch costs | 2.97 | 1.77 | 1.68 | 0.103 |
| Car speed * Global switch costs | -1.32 | 0.41 | -0.33 | 0.747 |
| Car speed * RMA RT | -0.54 | 0.45 | -1.20 | 0.241 |
| Car speed * Age * RMA RT | 3.16 | 1.67 | 1.90 | 0.065 |
| Car speed * Age * BADS | -0.07 | 0.26 | -0.26 | 0.800 |
| Car speed * Age * Global switch costs | -2.38 | 1.45 | -1.64 | 0.110 |
| Car speed * Age * Local switch costs | -3.56 | 1.91 | -1.87 | 0.070 |
| View | -2.89 | 0.79 | -3.68 | 0.001 |
| View * Age | -1.27 | 2.05 | -0.62 | 0.540 |
| View * BADS | 0.42 | 0.15 | 2.76 | 0.010 |
| View * Local switch costs | 4.06 | 1.69 | 2.41 | 0.022 |
| View * Global switch costs | -0.59 | 0.38 | -1.55 | 0.132 |
| View * RMA RT | -0.16 | 0.41 | -0.39 | 0.701 |
| View * Age * RMA RT | 1.92 | 1.46 | 1.32 | 0.198 |
| View * Age * BADS | -0.38 | 0.24 | -1.60 | 0.121 |
| View * Age * Global switch cost | -0.90 | 1.29 | -0.70 | 0.487 |
| View * Age * Local switch cost | -3.20 | 1.83 | -1.75 | 0.090 |
| Both directions | -0.23 | 0.82 | -0.28 | 0.779 |
| Both directions * Age | -2.06 | 2.24 | -0.92 | 0.364 |
| Both directions * BADS | 0.36 | 0.16 | 2.27 | 0.030 |
| Both directions * Local switch costs | 5.10 | 1.69 | 3.02 | 0.005 |
| Both directions * Global switch costs | -1.73 | 0.39 | -0.44 | 0.662 |
| Both directions * RMA RT | -0.77 | 0.44 | -1.77 | 0.088 |
| Both directions * Age * RMA RT | 1.98 | 1.62 | 1.23 | 0.227 |
| Both directions * Age * BADS | -0.10 | 0.25 | -0.41 | 0.683 |
| Both directions * Age * Global switch costs | -0.63 | 1.41 | -0.45 | 0.656 |
| Both directions * Age * Local switch costs | -3.71 | 1.81 | -2.05 | 0.048 |
| Traffic density | -0.15 | 0.12 | -1.25 | 0.210 |
| Traffic density * Age | -0.18 | 0.34 | -0.52 | 0.602 |
| Traffic density * BADS | 0.02 | 0.02 | 0.81 | 0.417 |
| Traffic density * Local switch costs | 0.47 | 0.24 | 1.93 | 0.054 |
| Traffic density * Global switch costs | 0.01 | 0.05 | 0.24 | 0.808 |
| Traffic density * RMA RT | -0.12 | 0.06 | -2.02 | 0.043 |
| Traffic density * Age * RMA RT | 0.36 | 0.24 | 1.50 | 0.133 |
| Traffic density * Age * BADS | -0.01 | 0.04 | -0.38 | 0.706 |
| Traffic density * Age * Global switch costs | -0.35 | 0.21 | -1.66 | 0.096 |
| Traffic density * Age * Local switch costs | -0.53 | 0.28 | -1.93 | 0.054 |
| Distractors | -0.21 | 0.36 | -0.62 | 0.537 |
| Distractors * Age | -0.99 | 1.02 | -0.97 | 0.335 |
| Distractors * BADS | 0.01 | 0.07 | 0.16 | 0.875 |
| Distractors * Local switch costs | 0.84 | 0.76 | 1.11 | 0.269 |
| Distractors * Global switch costs | -0.13 | 0.17 | -0.76 | 0.439 |
| Distractors * Age * RMA RT | 1.06 | 0.74 | 1.43 | 0.154 |
| Distractors * Age * BADS | -0.007 | 0.11 | -0.07 | 0.946 |
| Distractors * Age * Global switch costs | -0.79 | 0.65 | -1.22 | 0.224 |
| Distractors * Age * Local switch costs | -0.92 | 0.86 | -1.07 | 0.283 |
| Age | 7.32 | 4.06 | 1.80 | 0.083 |
| BADS | -0.90 | 0.28 | -3.27 | 0.002 |
| Local switch costs | -8.80 | 3.08 | -2.86 | 0.007 |
| Global switch costs | 1.09 | 0.71 | 1.53 | 0.135 |

Supplementary Table S4. Results of simple effects LMMs run to determine the direction of the interaction from the significant interactions found in Table 5. Simple effects models were run using the following equation: TTI ~ Factor (one level only) + (1|participant) + (1|trial)

| *Factor* | *β* | *SE* | *t-value* | *p-value* |
| --- | --- | --- | --- | --- |
| Local switch costs – YAs | -2.76 | 2.50 | -1.11 | 0.279 |
| Local switch costs – OAs | 0.76 | 0.89 | 0.86 | 0.411 |
| Global switch costs – YAs | 0.487 | 0.57 | 0.86 | 0.397 |
| Global switch costs – OAs | 3.47 | 1.93 | 1.80 | 0.101 |
| RMA RT – YAs | 0.28 | 0.65 | 0.44 | 0.665 |
| RMA RT – OAs | -2.60 | 2.11 | -1.23 | 0.245 |
| Traffic density – fast RMA RTs | -0.07 | 0.02 | -2.80 | 0.005 |
| Traffic density – slow RMA RTs | -0.11 | 0.03 | -4.02 | 5.88x10^-5^ |
| Car speed – low BADS scores | -2.61 | 0.13 | -20.43 | <1x10^-6^ |
| Car speed – high BADS scores | -1.83 | 0.06 | -28.98 | <1x10^-6^ |
| View – low BADS scores | -1.46 | 0.15 | -9.72 | <1x10^-6^ |
| View – high BADS scores | -1.07 | 0.07 | -14.73 | <1x10^-6^ |
| View – small local switch costs | -0.84 | 0.09 | -9.76 | <1x10^-16^ |
| View – large local switch costs | -0.82 | 0.13 | -6.52 | 1.11x10^-10^ |
| Both directions – low BADS scores | 0.61 | 0.13 | 4.73 | 7.54x10^-6^ |
| Both directions – high BADS scores | 0.97 | 0.07 | 14.55 | <1x10^-6^ |
| Both directions – small local switch costs | 1.14 | 0.08 | 15.01 | <1x10^-16^ |
| Both directions – large local switch costs | 1.45 | 0.12 | 12.44 | <1x10^-16^ |
| Both directions -YAs with small local switch costs | 1.15 | 0.08 | 14.51 | <1x10^-16^ |
| Both directions – YAs with large local switch costs | 1.75 | 0.16 | 11.08 | <1x10^-16^ |
| Both directions – OAs with small local switch costs | 1.06 | 0.25 | 4.23 | 5.97x10^-5^ |
| Both directions – OAs with large local switch costs | 1.21 | 0.17 | 7.30 | 5.32x10^-13^ |

Linear model fit comparisons for Experiment 1

**Supplementary Table S5.** The model fit comparison for the LMM run on TTI in Experiment 1. We started with the most maximal model that we could run, and pruned the random slopes until a model converged. If this was not the simplest model then we would continue to prune random slopes until the best fitting model could be determined. Here the only model that converged was the model with no random slopes.

| Model | AIC | BIC | Converge? |
| --- | --- | --- | --- |
| TTI ~ RMA RT * (Age * car speed + Age * View + Age * Lane number + Age * Near/Far lane) + local switch costs * (Age * car speed + Age * View + Age * Lane number + Age * Near/Far lane ) + global switch cost * (Age * car speed + Age * View + Age * Lane number + Age * Near/Far lane ) + BADS * (Age * car speed + Age * View + Age * Lane number + Age * Near/Far lane ) + (1 \| participant) + (1 \| trial) | 4731.03 | 5002.88 | yes |
| TTI ~ RMA RT * (Age * car speed + Age * View + Age * Lane number + Age * Near/Far lane) + local switch costs * (Age * car speed + Age * View + Age * Lane number + Age * Near/Far lane ) + global switch cost * (Age * car speed + Age * View + Age * Lane number + Age * Near/Far lane ) + BADS * (Age * car speed + Age * View + Age * Lane number + Age * Near/Far lane ) + (1 + RMA RT \| participant) + (1 + RMA RT \| trial) | 4733.68 | 5026.05 | No |
| TTI ~ RMA RT * (Age * car speed + Age * View + Age * Lane number + Age * Near/Far lane) + local switch costs * (Age * car speed + Age * View + Age * Lane number + Age * Near/Far lane ) + global switch cost * (Age * car speed + Age * View + Age * Lane number + Age * Near/Far lane ) + BADS * (Age * car speed + Age * View + Age * Lane number + Age * Near/Far lane ) + (1 + RMA RT + car speed \| participant) + (1 + RMA RT + car speed\| trial) | 4688.88 | 5012.02 | No |
| TTI ~ RMA RT * (Age * car speed + Age * View + Age * Lane number + Age * Near/Far lane) + local switch costs * (Age * car speed + Age * View + Age * Lane number + Age * Near/Far lane ) + global switch cost * (Age * car speed + Age * View + Age * Lane number + Age * Near/Far lane ) + BADS * (Age * car speed + Age * View + Age * Lane number + Age * Near/Far lane ) + (1 + RMA RT + car speed + View\| participant) + (1 + RMA RT + car speed + View\| trial) | 4663.85 | 5028.03 | No |
| TTI ~ RMA RT * (Age * car speed + Age * View + Age * Lane number + Age * Near/Far lane) + local switch costs * (Age * car speed + Age * View + Age * Lane number + Age * Near/Far lane ) + global switch cost * (Age * car speed + Age * View + Age * Lane number + Age * Near/Far lane ) + BADS * (Age * car speed + Age * View + Age * Lane number + Age * Near/Far lane ) + (1 + RMA RT + car speed + View + Near/Far lane\| participant) + (1 + RMA RT + car speed + View + Near/Far lane\| trial) | 4675.83 | 5065.66 | No |
| TTI ~ RMA RT * (Age * car speed + Age * View + Age * Lane number + Age * Near/Far lane) + local switch costs * (Age * car speed + Age * View + Age * Lane number + Age * Near/Far lane ) + global switch cost * (Age * car speed + Age * View + Age * Lane number + Age * Near/Far lane ) + BADS * (Age * car speed + Age * View + Age * Lane number + Age * Near/Far lane ) + (1 + RMA RT + car speed + View + Near/Far lane + lane number\| participant) + (1 + RMA RT + car speed + View + Near/Far lane + lane number\| trial) | 4697.83 | 5144.08 | No |
| TTI ~ RMA RT * (Age * car speed + Age * View + Age * Lane number + Age * Near/Far lane) + local switch costs * (Age * car speed + Age * View + Age * Lane number + Age * Near/Far lane ) + global switch cost * (Age * car speed + Age * View + Age * Lane number + Age * Near/Far lane ) + BADS * (Age * car speed + Age * View + Age * Lane number + Age * Near/Far lane ) + (1 + RMA RT + car speed + View + Near/Far lane + lane number + Age\| participant) + (1 + RMA RT + car speed + View + Near/Far lane + lane number + Age \| trial) | 4722.14 | 5235.07 | No |
| TTI ~ RMA RT * (Age * car speed + Age * View + Age * Lane number + Age * Near/Far lane) + local switch costs * (Age * car speed + Age * View + Age * Lane number + Age * Near/Far lane ) + global switch cost * (Age * car speed + Age * View + Age * Lane number + Age * Near/Far lane ) + BADS * (Age * car speed + Age * View + Age * Lane number + Age * Near/Far lane ) + (1 + RMA RT + car speed + View + Near/Far lane + lane number + Age + local switch costs \| participant) + (1 + RMA RT + car speed + View + Near/Far lane + lane number + Age + local switch costs\| trial) | 4747.00 | 5336.87 | No |
| TTI ~ RMA RT * (Age * car speed + Age * View + Age * Lane number + Age * Near/Far lane) + local switch costs * (Age * car speed + Age * View + Age * Lane number + Age * Near/Far lane ) + global switch cost * (Age * car speed + Age * View + Age * Lane number + Age * Near/Far lane ) + BADS * (Age * car speed + Age * View + Age * Lane number + Age * Near/Far lane ) + (1 + RMA RT + car speed + View + Near/Far lane + lane number + Age + local switch costs + global switch costs \| participant) + (1 + RMA RT + car speed + View + Near/Far lane + lane number + Age + local switch costs + global switch costs\| trial) | 4777.50 | 5454.57 | No |
| TTI ~ RMA RT * (Age * car speed + Age * View + Age * Lane number + Age * Near/Far lane) + local switch costs * (Age * car speed + Age * View + Age * Lane number + Age * Near/Far lane ) + global switch cost * (Age * car speed + Age * View + Age * Lane number + Age * Near/Far lane ) + BADS * (Age * car speed + Age * View + Age * Lane number + Age * Near/Far lane ) + (1 + RMA RT + car speed + View + Near/Far lane + lane number + Age + local switch costs + global switch costs + BADS \| participant) + (1 + RMA RT + car speed + View + Near/Far lane + lane number + Age + local switch costs + global switch costs + BADS\| trial) | 4815.02 | 5589.55 | No |

Linear model fit comparisons for Experiment 2.

**Supplementary Table S6.** The model fit comparison for the LMM run on TTI in Experiment 2. We started with the most maximal model that we could run, and pruned the random slopes until a model converged. If this was not the simplest model then we would continue to prune random slopes until the best fitting model could be determined. Here the only model that converged was the model with random intercepts for participants and trials, and random slopes for age group, car speed, and the direction the cars travelled from

| Model | AIC | BIC | Converge? | Notes |
| --- | --- | --- | --- | --- |
| TTI ~ RMA RT * (age * car speed + age * traffic + age * direction + age * distractors) + BADS * (age * car speed + age * traffic + age * direction + age * distractors) + global switch cost * (age * car speed + age * traffic + age * direction + age * distractors) + Local switch cost * (age * car speed + age * traffic + age * direction + age * distractors) + (1 \| participant) + (1 \| trial) | 34047.73 | 34415.12 | No |  |
| TTI ~ RMA RT * (age * car speed + age * traffic + age * direction + age * distractors) + BADS * (age * car speed + age * traffic + age * direction + age * distractors) + global switch cost * (age * car speed + age * traffic + age * direction + age * distractors) + Local switch cost * (age * car speed + age * traffic + age * direction + age * distractors) + (1 + Age \| participant) + (1 + Age \| trial) | 34055.71 | 34450.77 | No |  |
| TTI ~ RMA RT * (age * car speed + age * traffic + age * direction + age * distractors) + BADS * (age * car speed + age * traffic + age * direction + age * distractors) + global switch cost * (age * car speed + age * traffic + age * direction + age * distractors) + Local switch cost * (age * car speed + age * traffic + age * direction + age * distractors) + (1 + Age + Speed \| participant) + (1 + Age + Speed \| trial) | 34005.71 | 34442.35 | No |  |
| TTI ~ RMA RT * (age * car speed + age * traffic + age * direction + age * distractors) + BADS * (age * car speed + age * traffic + age * direction + age * distractors) + global switch cost * (age * car speed + age * traffic + age * direction + age * distractors) + Local switch cost * (age * car speed + age * traffic + age * direction + age * distractors) + (1 + Age + Speed + Direction \| participant) + (1 + Age + Speed + Direction \| trial) | 33256.70 | 33887.41 | Yes |  |
| TTI ~ RMA RT * (age * car speed + age * traffic + age * direction + age * distractors) + BADS * (age * car speed + age * traffic + age * direction + age * distractors) + global switch cost * (age * car speed + age * traffic + age * direction + age * distractors) + Local switch cost * (age * car speed + age * traffic + age * direction + age * distractors) + (1 + Age + Speed + Direction + Traffic \| participant) + (1 + Age + Speed + Direction + Traffic \| trial) | 33247.28 | 33961.16 | No |  |
| TTI ~ RMA RT * (age * car speed + age * traffic + age * direction + age * distractors) + BADS * (age * car speed + age * traffic + age * direction + age * distractors) + global switch cost * (age * car speed + age * traffic + age * direction + age * distractors) + Local switch cost * (age * car speed + age * traffic + age * direction + age * distractors) + (1 + Age + Speed + Direction + Traffic + RMA RT \| participant) + (1 + Age + Speed + Direction + Traffic + RMA RT \| trial) | 33270.27 | 34081.19 | No |  |
| TTI ~ RMA RT * (age * car speed + age * traffic + age * direction + age * distractors) + BADS * (age * car speed + age * traffic + age * direction + age * distractors) + global switch cost * (age * car speed + age * traffic + age * direction + age * distractors) + Local switch cost * (age * car speed + age * traffic + age * direction + age * distractors) + (1 + Age + Speed + Direction + Traffic + RMA RT + global switch costs + local switch costs \| participant) + (1 + Age + Speed + Direction + Traffic + RMA RT + global switch costs + local switch costs \| trial) | 33332.56 | 34379.13 | No |  |
| TTI ~ RMA RT * (age * car speed + age * traffic + age * direction + age * distractors) + BADS * (age * car speed + age * traffic + age * direction + age * distractors) + global switch cost * (age * car speed + age * traffic + age * direction + age * distractors) + Local switch cost * (age * car speed + age * traffic + age * direction + age * distractors) + (1 + Age + Speed + Direction + Traffic + RMA RT + global switch costs + local switch costs + distractors \| participant) + (1 + Age + Speed + Direction + Traffic + RMA RT + global switch costs + local switch costs + distractors \| trial) |  |  |  | Computer crashed before completion |
